# Approaches to Investigate Selective Dietary Polysaccharide Utilization by Human Gut Microbiota at a Functional Level

**DOI:** 10.3389/fmicb.2021.632684

**Published:** 2021-02-19

**Authors:** Leeann Klassen, Xiaohui Xing, Jeffrey P. Tingley, Kristin E. Low, Marissa L. King, Greta Reintjes, D. Wade Abbott

**Affiliations:** ^1^Lethbridge Research and Development Centre, Agriculture and Agri-Food Canada, Lethbridge, AB, Canada; ^2^Department of Chemistry and Biochemistry, University of Lethbridge, Lethbridge, AB, Canada; ^3^Max Planck Institute for Marine Microbiology, Bremen, Germany

**Keywords:** microbiome, carbohydrate, carbohydrate-active enzyme, phylogeny, next-generation physiology, carbohydrate probe, fluorescent polysaccharides

## Abstract

The human diet is temporally and spatially dynamic, and influenced by culture, regional food systems, socioeconomics, and consumer preference. Such factors result in enormous structural diversity of ingested glycans that are refractory to digestion by human enzymes. To convert these glycans into metabolizable nutrients and energy, humans rely upon the catalytic potential encoded within the gut microbiome, a rich collective of microorganisms residing in the gastrointestinal tract. The development of high-throughput sequencing methods has enabled microbial communities to be studied with more coverage and depth, and as a result, cataloging the taxonomic structure of the gut microbiome has become routine. Efforts to unravel the microbial processes governing glycan digestion by the gut microbiome, however, are still in their infancy and will benefit by retooling our approaches to study glycan structure at high resolution and adopting next-generation functional methods. Also, new bioinformatic tools specialized for annotating carbohydrate-active enzymes and predicting their functions with high accuracy will be required for deciphering the catalytic potential of sequence datasets. Furthermore, physiological approaches to enable genotype-phenotype assignments within the gut microbiome, such as fluorescent polysaccharides, has enabled rapid identification of carbohydrate interactions at the single cell level. In this review, we summarize the current state-of-knowledge of these methods and discuss how their continued development will advance our understanding of gut microbiome function.

## Introduction

Human diets contain a vast diversity of complex carbohydrates; yet the human genome encodes only 17 known digestive enzymes to digest lactose, starch, and sucrose (El Kaoutari et al., 2013). To compensate for this catalytic deficiency, humans rely on their gut microbiome – a dynamic community of bacteria, fungi, protozoa, and viruses – which encodes proteins designed to sense and consume dietary glycans. The gut microbiome also helps stimulate host immunity, regulate pathogen growth (e.g., competitive exclusion), synthesize essential amino acids, and provides other health outcomes (Krajmalnik-Brown et al., 2012; Honda and Littman, 2016; Gentile and Weir, 2018; Li et al., 2018; Valdes et al., 2018), including improving responsiveness to chemotherapy (Mager et al., 2020). Cultural habits (Sonnenburg and Sonnenburg, 2019; Ozkul et al., 2020), seasonal variability (Smits et al., 2017), geography (De Filippo et al., 2010), and individual lifestyle preferences (Dahl et al., 2020; Pittman, 2020) influence the structure, function, and diversity of the gut microbiome. This is demonstrated by the striking differences in microbiome composition between hunter-gatherer and agricultural societies; societies that typically consume diets with high and low levels of microbial accessible carbohydrates, respectively (Jha et al., 2018; Sonnenburg and Sonnenburg, 2019). Although easy to determine, microbial composition alone provides little insight into the mechanisms that govern taxonomic shifts, and deciphering glycan-microbe interactions requires advanced -omic techniques. In this review, we highlight research methods to: (i) characterize the chemical structure of carbohydrates in personalized diets; (ii) define the catalytic potential of gut microbiomes using high-throughput sequencing and improve *in silico* tools to more accurately decode functional information contained within sequence datasets; and (iii) develop next-generation physiology approaches that enable the rapid identification of active microbial cells to verify the functional predictions emerging from sequencing data. These advanced techniques have and will continue to accelerate our understanding of carbohydrate utilization in the gut, and ultimately, determine how diet shapes the structure, function, and diversity of the gut microbiome.

## Profiling the Chemical Structure of Carbohydrates in Food

### Structural Diversity of Complex Carbohydrates in Human Food

**FIGURE 1:**
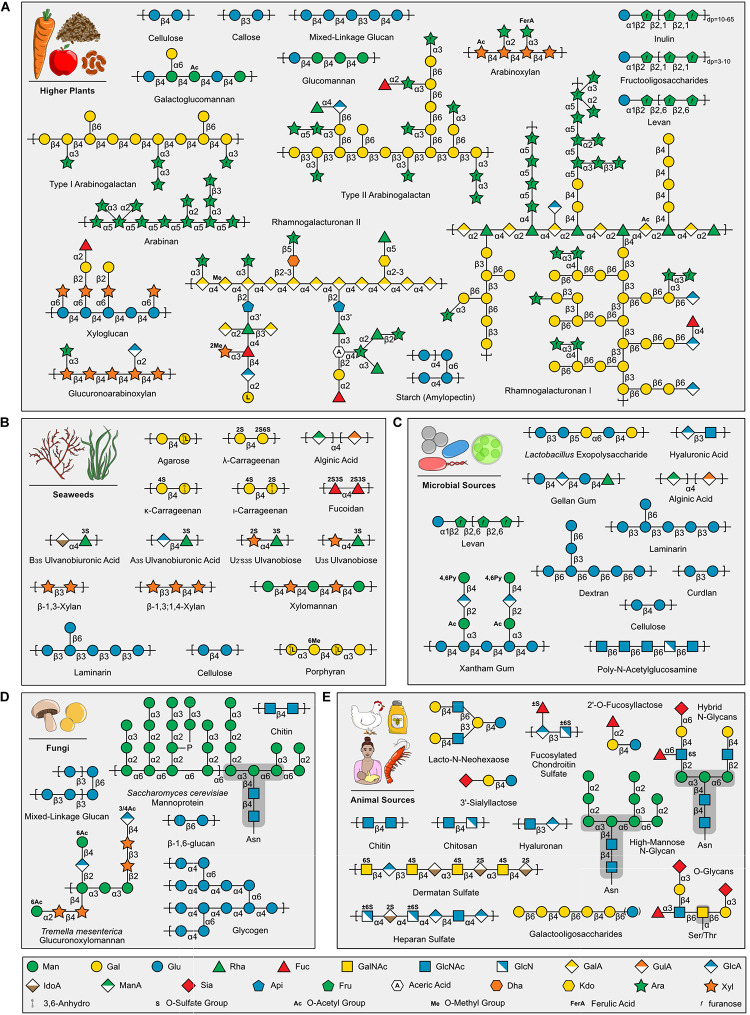
Examples of carbohydrates in human foods grouped by source. **(A)** Higher plants **(B)** Seaweeds **(C)** Microbial Sources **(D)** Fungi **(E)** Animal Sources, including Human Milk Oligosaccharides (e.g., 2′-O-Fucosyllactose, 3′-Sialyllactose, and Lacto-N-Neohexaose). Carbohydrate symbols follow the Symbol Nomenclature for Glycans guidelines (Varki et al., 2015; Neelamegham et al., 2019). Note, glycogen is displayed in **(D)**, but is also found in **(C,E)** (not shown). *Saccharomyces cerevisiae* mannoprotein (Cuskin et al., 2015) and *Tremella mesenterica* glucuronoxylomannan (Vinogradov et al., 2004) selected as examples of fungal carbohydrate diversity. Asparagine (Asn) and Serine/Threonine (Ser/Thr) protein link shown for N- and O-glycans, respectively, and core structures highlighted with dark gray background. Sialic acids are a diverse group of nine carbon sugars with varying levels and positions of acetylation depending on the animal source. Importantly, Figure 1 highlights representative carbohydrates found in different food groups and is intended to demonstrate the structural diversity present within food; other excellent reviews exist that provide comprehensive structural information for each group [e.g., Pectins (Saffer, 2018), Human Milk Oligosaccharides (Oliveira et al., 2015)].

Human diets contain structurally diverse carbohydrates that vary between food groups (Figure 1 and Supplementary Table 1). Plant-based foods (e.g., cereals, legumes, vegetables, fruits, roots, tubers; Figure 1A) are composed up to 50–70% of glycans, dry weight, that are present as storage polysaccharides (e.g., starch and fructans) and cell wall structural polysaccharides (e.g., cellulose, hemicelluloses, and pectins) (Burton and Fincher, 2012; Padayachee et al., 2017; Saffer, 2018; Anderson and Kieber, 2020; Marcotuli et al., 2020). Moreover, glycans are produced as exudates and mucilage used as gums in food industries (e.g., gum arabic, guar gum, konjac gum) (Mirhosseini and Amid, 2012; Xing et al., 2017; Yu et al., 2017). Glycogen is a common storage polysaccharide for microbial (Figure 1C), fungal (Figure 1D), and animal (Figure 1E) cells and is the major glycan in meats (Mellor et al., 1958; Fernandez and Tornberg, 1991; Komatsu et al., 2014). Many foods and beverages contain a complex mixture of polysaccharides, oligosaccharides, and monosaccharides from multiple biological sources (Saha and Bhattacharya, 2010; Sivam et al., 2010); whereas, some products, such as milk, honey, beer, wine, and maple syrup, have a simpler carbohydrate profile, being primarily composed of oligosaccharides (e.g., fructooligosaccharides, rhamnogalacturonan II, sialyllactose) with various chemistries and degrees of polymerization (dp) (Figure 1E; Morales et al., 2006; Doco et al., 2015; Oliveira et al., 2015; Kanyer et al., 2017; Sato et al., 2019). More specialized food groups, such as edible seaweeds (Figure 1B), bacteria and microalgae (Figure 1C), fungi (Figure 1D), and seafood (Figure 1E), present structurally unique polysaccharides that have a range of bioactivities within the host (Gow et al., 2017; Pandya et al., 2019; Wu et al., 2019) and are also used commercially as functional food ingredients and additives (Usov, 2011; Forján et al., 2014; Schmid et al., 2015; Villarruel-López et al., 2017; Pangestuti and Arifin, 2018; Thinh et al., 2018; Ustyuzhanina et al., 2018; Alba and Kontogiorgos, 2019; Cherry et al., 2019; Hsieh and Harris, 2019; Kidgell et al., 2019; Praveen et al., 2019; Becker et al., 2020; Osemwegie et al., 2020; Xiong et al., 2020). The wide array of structurally diverse food carbohydrates not absorbed by the host become substrates for the human gut microbiome.

It is common knowledge that dietary fiber is good for digestive health and the abundance of available supplements, such as Metamucil^®^ derived from the *Plantago ovata* seed husks, reflect this. However, deciphering which carbohydrates elicit beneficial effects on the gut microbiome and the mechanisms behind these effects are less understood. The prebiotic effect is one of most well studied aspects of dietary carbohydrate bioactivity. Prebiotics are defined as “substrate[s] that [are] selectively utilized by host microorganisms conferring a health benefit” (Gibson et al., 2017). Beneficial bacterial species, such as those belonging to the *Lactobacillus* and *Bifidobacterium* genera, will ferment these prebiotics and produce short-chain fatty acids. Selectively feeding these species can improve host gastrointestinal health through pathogen exclusion, increased expression of tight junction proteins, and downregulation of inflammation (Plaza-Díaz et al., 2017; La Fata et al., 2018; Monteagudo-Mera et al., 2019). These beneficial health outcomes can begin to take place immediately after birth. The consumption of human milk oligosaccharides in breast milk, such as 3′sialyllactose and fucosyllactose (Figure 1E), promote the growth of beneficial species, such as *Bifidobacterium* spp. (Gnoth et al., 2000; LoCascio et al., 2007). As humans physically develop and transition to solid food, sources of prebiotics can be found in foods such as fruits, vegetables, and cereal and pulse crops.

Among the currently accepted sources of prebiotics, inulin-type fructans and fructooligosaccharides (Figure 1A) are the most studied and frequently consumed (Flamm et al., 2001; Davani-Davari et al., 2019). These prebiotics naturally occur in chicory, onions, asparagus, garlic, and bananas. As the commercial landscape for prebiotics continues to develop, the term “candidate” prebiotics is being used for carbohydrates (e.g., pectin, xylo-oligosaccharides, and β-glucan) that show potential prebiotic effects *in vitro* or in animal experiments, but lack sufficient data from human studies (Scott et al., 2020). Monosaccharide chemistry, glycosidic linkage, chemical modification, dp, and degree and site of branching that exists within the structure of prebiotic carbohydrates ultimately determines which microbial species have the genetic tools to utilize the substrate as an energy source (Scott et al., 2014). Therefore, defining the fine-chemistry of complex carbohydrates in foods and candidate prebiotics is a pivotal step to elucidating their effect on the microbiome.

### Current Glycomics Methods to Study Food Carbohydrates

Different glycomic methods are required to study unique structural features of carbohydrates, such as linkage, monosaccharide content, or dp, and selecting the appropriate preparative and analytical method needs to be carefully considered (Tingley et al., 2021). Dietary polysaccharide analysis begins with hot water extraction from source material, followed by sequential fractionation by preparative column chromatography, solvent extraction, and ethanol precipitation. Some glycomic analyses (e.g., glycosidic linkage), however, are conducted on unfractionated food polysaccharide mixtures (Usov, 2011; Xing et al., 2013, 2014, 2015b; Yu et al., 2017; Wood et al., 2018; Li et al., 2019; Xiong et al., 2020). Dietary oligosaccharides are typically analyzed after crude extraction using water or ethanol solutions and purification by solid phase extraction (Jaeger et al., 2017; Xing et al., 2017; Roberts et al., 2018; Little et al., 2019). Ultraviolet-visible spectrophotometry can be used to colorimetrically quantify total carbohydrates in food samples (e.g., total starch in a plant, total glycogen in meat) (Xing et al., 2009, 2013, 2014). Additionally, monosaccharide composition of food carbohydrates can be determined using high-performance anion exchange chromatography coupled to pulsed amperometric detection (HPAEC-PAD), reversed-phase high-performance liquid chromatography coupled to ultraviolet-visible spectroscopy (RP-HPLC-UV), or gas chromatography coupled to mass spectrometry or flame ionization detection (GC-MS/FID) (Usov, 2011; Xing et al., 2014, 2015b, 2017; Roberts et al., 2018; Little et al., 2019; Xiong et al., 2020).

Several methods exist to provide a more detailed understanding of carbohydrate structures. For example, GC-MS/FID is a preeminent tool for linkage analysis (methylation analysis) of food carbohydrates (Usov, 2011; Xing et al., 2013, 2014; Jaeger et al., 2017; Yu et al., 2017; Roberts et al., 2018; Wood et al., 2018; Li et al., 2019; Little et al., 2019). While HPAEC-PAD is a conventional tool for the analysis of food oligosaccharide composition, high-resolution mass spectrometry (e.g., MALDI-TOF-MS, LC-ESI-MS/MS) using MS and tandem MS (MS/MS) acquisition modes are becoming common methods to determine the accurate mass and linkage sequence of food oligosaccharides (Xing et al., 2015a; Jaeger et al., 2017; Roberts et al., 2018; Little et al., 2019). Notably, recently developed ESI-MS/MS fragmentation methods (e.g., charge transfer dissociation) have proven powerful for the detailed structural characterization of natural complex oligosaccharides, such as mixed linkage glucan and sulfated anions (Ropartz et al., 2016, 2017; Buck-Wiese et al., 2020). High-performance size-exclusion chromatography (HPSEC) systems coupled to multiple detectors including refractive index (RI) detector, viscometer, and multiple-angle laser light scattering detectors are used to determine molecular weight, size, intrinsic viscosity, and conformational parameters of dietary polysaccharides (Xing et al., 2013, 2014, 2015b; Yu et al., 2017). The detailed structural features of food carbohydrates can be further elucidated using solution-state ^1^H and ^13^C NMR spectroscopy (Xing et al., 2014, 2015a,b, 2017; Jaeger et al., 2017). Fourier-transform infrared spectroscopy (FTIR) is used for non-destructive characterization of food carbohydrates, and the determination of the degree of substitution of polysaccharides (Duarte et al., 2002; Xing et al., 2014, 2015b). Glycomic approaches can be used to define the structure of carbohydrates before (substrate) and after (products) interaction with the gut microbiome, providing higher-level insights into the microbial processes governing its metabolic function.

## Decoding the Catalytic Potential of Gut Microbiomes

### Carbohydrate-Active Enzymes

The human gut microbiome encodes a wealth of carbohydrate-active enzymes (CAZymes) designed for the biosynthesis and modification of glycans and their derivatives, as well as the saccharification of dietary glycans to promote the growth of metabolically capable microbes. CAZymes have evolved to accommodate the diversity of monosaccharide composition, stereochemical linkage, and branching of dietary glycans (Figure 1). CAZymes are categorized into five classes: glycoside hydrolases (GHs), polysaccharide lyases (PLs), carbohydrate esterases (CEs), auxiliary activities (AAs), and glycosyltransferases (GTs) (Lombard et al., 2014). GHs, PLs, and CEs play important roles in the digestion of carbohydrates by the human gut microbiome. GHs depolymerize carbohydrate substrates by hydrolyzing glycosidic linkages, and are composed of 168 sequence-related families (CAZY, 2021). Microorganisms depend on these enzymes to saccharify and metabolize polysaccharides in the human diet, such as starch (Tancula et al., 1992) and complex pectins, such as rhamnogalacturonan II [which can contain up to 21 distinct linkages (Ndeh et al., 2017)]. PLs act by β-elimination to cleave uronic acid containing carbohydrates, such as pectin backbone (Figure 1A), alginic acid (Figure 1C), and heparan sulfate (Figure 1E). CEs cleave carbohydrate esters, such as *N*-acetylglucosamine (GlcNAc) found in microbial, fungal, and animal food groups.

**FIGURE 2:**
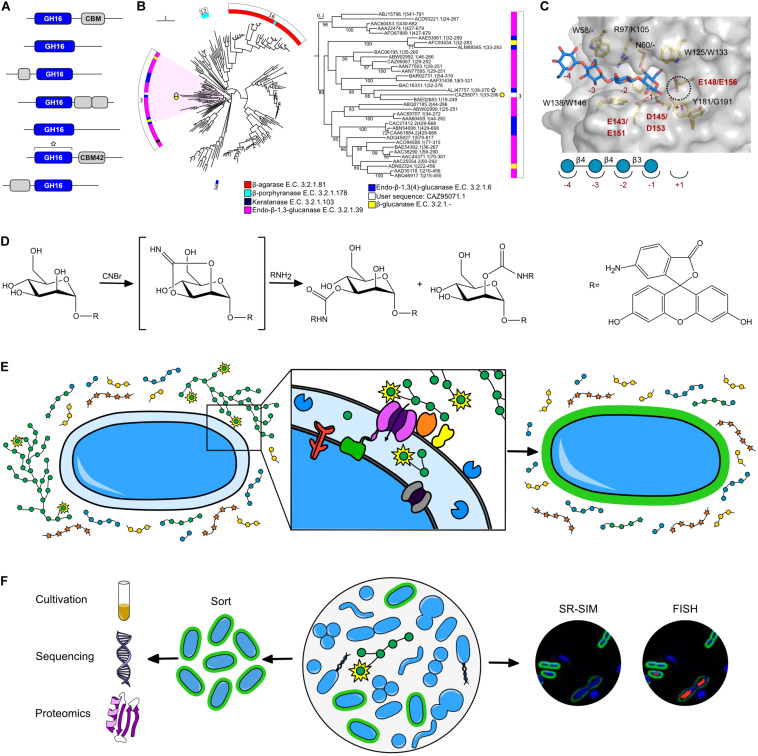
Current *in silico* and NGP tools to study microbiome-carbohydrate interactions. **(A)** Hypothetical sequence dataset of GH16 enzymes, including a representative user sequence (white star). **(B)** GH16 family tree produced by SACCHARIS (Jones et al., 2018) using characterized GH16 members and the representative uncharacterized user sequence found in A. Tree analyzed using the Interactive Tree of Life (iTOL) (Letunic and Bork, 2019). Inner ring maps the E.C. # activity of sequences, while the outer ring denotes sequence subfamily. The clade that includes the user sequence is highlighted and expanded in the right panel. Sequence labels denote accession numbers and dbCAN predicted domain boundaries. **(C)** Three-dimensional structure of a closely related GH16 homolog (ALJ47757.1, PDB code 5NBO (Tamura et al., 2017). The active site residues (yellow) and carbohydrate product (blue) are displayed as sticks; the semi-transparent solvent accessible surface of the protein is shown in gray. Homologous residues from the input sequence in 1B are labeled (5NBO/User sequence). Conserved catalytic residues (E143/E151 nucleophile; E148/156 acid/base; D145/D153 electrostatic “helper”) in red to highlight absolute conservation based on primary sequence alignment, suggesting User enzyme has similar activity as the GH16 homolog. **(D)** Proposed chemical reaction of fluoresceinamine conjugation to a monosaccharide residue (e.g., mannose), resulting in a FLA-PS probe (Glabe et al., 1983). R = fluoresceinamine, shown on right. **(E)** Schematic of FLA-PS uptake into the periplasm of a gram-negative bacterial cell. Monosaccharides labeled with fluoresceinamine indicated by yellow star around residue. Expanded panel shows binding of FLA-PS by outer membrane binding proteins (orange and yellow), transport of FLA-PS into the cell by transport protein (purple), and further saccharification of FLA-PS by GHs (blue). Other proteins involved in pathway include regulator (red), TonB complex (green), and inner membrane transporter (gray). **(F)** Schematic of FLA-PS incubation in a complex community and predicted downstream applications, including cultivation and omics analysis (left) and microscopy (right). Green = FLA-PS stained cells. Blue = DNA co-stain (DAPI). Red = 16S rRNA FISH probe.

The CAZy database, established in 1999, is responsible for the curation of CAZyme classes and families (Lombard et al., 2014) and has provided a digital framework for CAZyme annotation (Zhang et al., 2018). CAZymes are grouped into families that have conserved tertiary structures, mechanisms, and catalytic residues; however, this does not necessarily translate into redundant functions. In polyspecific families, members partition into groups termed “subfamilies” that can differ in their mode of action (e.g., *endo*- verses *exo*-hydrolysis) or substrate specificity (Stam et al., 2006; St John et al., 2010; Aspeborg et al., 2012; Mewis et al., 2016). For example, the GH16 family is currently divided into 27 subfamilies active on β-glucans, xyloglucans, and β-galactans (Figure 2B; Viborg et al., 2019). The presence or abundance of these subfamilies within the genome of an organism can be highly reflective of its metabolic response to geospatially restricted diets: GH16 subfamily 3, found to be distributed throughout the globe, is primarily active on β-1,3 and β-1,3(4)-glucans that can be found in cereal polysaccharides (Tamura et al., 2017), whereas in Asian cultures, human gut *Bacteroides* spp. have acquired GH16 subfamily 12 (i.e., porphyranase) and 16 (i.e., agarase) members from red algal microbiome species through lateral gene transfer (Hehemann et al., 2012; Pluvinage et al., 2018). Furthermore, GH16 members from *Bacteroides* spp. have been shown to be upregulated when grown on human milk oligosaccharides (Marcobal et al., 2011), and recent studies have demonstrated GH16 members which prefer milk oligosaccharides to mucin oligosaccharides (Crouch et al., 2020). However, more research needs to be done to determine the phylogenetic structure-function relationships of these novel GH16s and to determine their prevalence in infant vs. adult microbiota.

Not all polyspecific families have been divided into subfamilies. This is the case for GH92s, which have been shown to be tailored for saccharification of α-mannans in fungal polysaccharides and human glycans, with members tailored for α-1,2, α-1,3, α-1,4, α-1,6 linked and α-1-mannosyl-phosphate substrates (Zhu et al., 2010; Cuskin et al., 2015). The absence of defined subfamilies can be a challenge for the prediction of CAZyme activity and further characterization, which can be alleviated by detailed inspection of phylogenetic relationships that provide further insight into the specificity of uncharacterized CAZymes.

### Methods for Decoding Uncharacterized CAZyme Function

*In silico* tools for CAZyme curation, annotation, and phylogenetic comparison have aided in the functional prediction of the consistently expanding -omic and meta-omic (i.e., genomic, transcriptomic, proteomic) sequence space. Gene annotation tools and databases have expanded with the growth of -omic datasets, bringing with them their own advancements and limitations (Salzberg, 2019; Lobb et al., 2020). However, novel and polyspecific families continue to convolute functional CAZyme annotation. Integrated software tools and online resources have come online to enable users to analyze data within the CAZy framework, including dbCAN for the annotation of uncharacterized CAZyme modules (Figure 2A). Multiple tools have been generated originating from dbCAN annotations, such as: PULpy (Stewart et al., 2018) and DRAM (Shaffer et al., 2020) for the prediction and annotation of polysaccharide catabolism, and SACCHARIS (Figure 2B; Jones et al., 2018) and CUPP (Barrett and Lange, 2019) for high-resolution phylogenetic analyses within CAZyme families. CAZyme phylogenies are used to determine the similarity of uncharacterized user sequences to characterized CAZyme sequences. These peptide- and protein-based sequence alignments can help to elucidate the catalytic residues based their relatedness to previously characterized enzymes, as represented in Figure 2C which shows absolute conservation of catalytic residues between a user sequence and characterized GH16, and strengthens the prediction that these enzymes have a similar activity. Furthermore, the analysis of the entire set of CAZymes encoded in a bacterial genome (i.e., CAZome) can provide insight into the catalytic potential of individual populations within the gut microbiome (Jones et al., 2018). Although the development of *in silico* tools has increased the throughput of parsing -omics data, predicted functions are still hypothetical and often rely on the level of relatedness between user sequences and CAZymes with defined catalytic specificities. Biochemical characterization is the final attributor of function, and continued efforts and development of platforms to discover new enzyme specificities will improve the accuracy of *in silico* pipelines to predict CAZyme function from sequence datasets (Helbert et al., 2019).

## Next-Generation Physiology to Identify the Function of Single Cells

### Visualizing Microbiome-Carbohydrate Interactions With Carbohydrate Probes

Although the accuracy and ease of functional gene prediction is rapidly improving, advances in functional assignment of microbial-glycan interactions have been limited. This is because cultivation can be difficult and low throughput (Alain and Querellou, 2009; Börner, 2016), even with the development of culturomic techniques [e.g., iChip (Berdy et al., 2017)]. Recently, phenotype-based methods have gained popularity because they enable functional screening of microbial cells in complex microbial communities. Furthermore, they can be non-destructive, thereby facilitating single-cell isolation for downstream characterization in an approach called next-generation physiology (NGP) (Hatzenpichler et al., 2020). Carbohydrates have physical properties that make them difficult to detect analytically. Derivatization alters the physical characteristics of glycans to facilitate analytical tracing of these substrates *in vivo* and *in situ*. Recent advances in carbohydrate probe design, together with contemporary microbiological techniques, such as Raman microspectroscopy (Lee et al., 2019), bioorthogonal non-canonical amino acid tagging (BONCAT) (Glenn et al., 2017; Reichart et al., 2020), and fluorescence *in situ* hybridization (FISH) (Bayani and Squire, 2004), have led to paradigm shifts in how microbial-glycan interaction can be studied.

Most carbohydrate probes are produced by isotope or chemical labeling. Historically, radioactive and stable isotope probes have been used to characterize cellular function, most commonly with isotopic monosaccharide and oligosaccharides that are commercially prepared (Wagner, 2009). Importantly, isotope labels do not alter the size or structure of the carbohydrate, which makes them ideal for studying metabolic pathways involving transporters and enzymes that have strict recognition determinants. More recently, a Raman-based cell sorting approach was developed to extend the use of stable isotope probes to isolate metabolically active cells (Lee et al., 2019). Selective isolation of single cells enables downstream cultivation and single-cell genomics to identify the target bacteria. Additionally, these labels have been used alongside FISH probes to taxonomically identify active cells (Li et al., 2008). Although stable isotope labeling is valuable, widespread use has been limited due to restrictions around isotope work, radioactive waste produced, and the highly specialized equipment necessary for analysis (e.g., microautoradiography and Raman microspectroscopy).

Relative to isotopic labels, chemical labels are often less expensive, more easily detected, and include a wider variety of adducts that can be used simultaneously to create multi-colored images. Several strategies exist to conjugate a carbohydrate to a chemical adduct. One example is click chemistry, which is a unique approach to study biomolecules in living systems (Kolb et al., 2001). Bioorthogonal click chemistry is used to conjugate a modified substrate, most commonly through an azide reaction, to a probe, such as a fluorophore. The reaction rapidly produces a detectable signal that can be used to isolate the substrate. Unfortunately, most azide click reactions rely on a copper catalyst, which is highly toxic to microbes, although some work has been done to mitigate this detrimental side effect (Zhang and Zhang, 2013). Despite this limitation, copper-catalyzed alkyne-azide click chemistry has been shown to successfully label living cells (Hong et al., 2010). More recently, this technique was extended for the *in vivo* visualization of bacteria in the mouse gut microbiome (Wang et al., 2020).

Another commonly used chemical labeling method to study carbohydrates in biological systems is fluorescence-based labeling (Yan et al., 2015), for example, boronic acid-based fluorescence detection, fluorogenic labeling agents, fluorescently labeled glycolipids, fluorescence monosaccharide derivatives or analogs (Tao et al., 2019), and fluorescently labeled polysaccharides (Arnosti, 2003). Fluorescent monosaccharide probes, such as the glucose analog 2-NBDG, have been around for some time, but have only recently been used to detect bacteria in the rumen microbiome that uptake and metabolize glucose (Tao et al., 2019). Use of 2-NBDG and similar monosaccharide probes is limited, however, because transport proteins are typically unable to accommodate the bulky fluorophore and transport it into the cell. In contrast, fluorescently labeled polysaccharides (FLA-PS) (Glabe et al., 1983) provide more flexibility to study substrate uptake, and have been very successful when fluoresceinamine is used as the conjugate fluorophore (Figure 2D; Reintjes et al., 2017; Hehemann et al., 2019; Klassen et al., 2021). In contrast to methods that only label the reducing end, using a stochastic method to label hydroxyl groups of monosaccharide residues generates a library of related probes that are sampled by enzymes and transporters (Figure 2E); probes that are amenable to importation can be visualized by fluorescence microscopy (Figure 2F). Fluoresceinamine derivatives are stable and exhibit activity equal to underivatized polysaccharides when used in inhibition assays (Glabe et al., 1983) with potential for bacteria-glycan research applications.

### FLA-PS in Gut Microbiome Research

Although FLA-PS have been used to study glycan metabolism in marine systems since the 1990s (Arnosti, 1995, 1996, 2003; Cuskin et al., 2015; Reintjes et al., 2017), studies in the gut microbiome have just commenced. In 2017, FLA-PS uptake was visualized for rhamnogalacturonan II and yeast mannan in the human gut symbiont, *Bacteroides thetaiotaomicron* (Hehemann et al., 2019). This study demonstrated that FLA-PS can be used to correlate metabolic phenotypes with genotypes and to differentiate between foraging strategies (Grondin et al., 2017). More recently, FLA-PS were successfully used in combination with FISH to detect and identify metabolically active cells in rumen community, respectively, and to quantify differences in glycan uptake rates between *Bacteroides* strains (Klassen et al., 2021).

Moving forward, FLA-PS has great potential to streamline the identification and isolation of metabolically active microorganisms in microbiome research. FLA-PS could be used to visualize spatial distribution and mobility of fluorescent carbohydrates in the gastrointestinal tract of animals using whole-body imaging systems or as a means to sort metabolically active cells by fluorescence-activated cell sorting (FACS) (Figure 2F). Sorted cells could be sequenced or cultivated for applications in enzyme discovery, probiotics, or synthetic microbiomes. In addition, if fluorescent conjugates can be found with different emission wavelengths, multiple FLA-PS glycans could be tracked simultaneously; a breakthrough that would allow researchers to visualize and study carbohydrate preferences of gut bacteria and study nutrient competition dynamics in complex microbial communities.

## Conclusion

Dietary glycans shape the structure, function, and diversity of the gut microbiome. New methods have provided a welcomed refresh to the toolkit researchers have to study glycan structure and determine how they interact with members of the gut microbiome. These recent technical advances will provide unprecedented insight into processes by which glycans are selectively consumed by bacterial populations within complex microbial communities. Combined use of glycomics, CAZyme bioinformatic tools, and chemical probes to study next-generation physiology approaches will be pivotal for deciphering sequence datasets and open a new frontier for prescribed use of glycans as drivers of microbiome function and human health.

## Author Contributions

LK wrote main section of the manuscript and prepared figures. XX wrote glycomics section and verified carbohydrate structures in Figure 1. JT wrote the CAZyme section and produced the GH16 tree for Figure 2B. KL generated the active site model for Figure 2C and edited the manuscript. MK provided the paragraph on prebiotics and edited the manuscript. GR wrote the FLA-PS section and edited the manuscript. DA was invited to contribute to the special issue, supervised the outline design, and assisted with figure and manuscript preparation and editing. All authors contributed to the article and approved the submitted version.

## Conflict of Interest

The authors declare that the research was conducted in the absence of any commercial or financial relationships that could be construed as a potential conflict of interest.

## References

[B1] Alain K. Querellou J. (2009). Cultivating the uncultured: limits, advances and future challenges. *Extremophiles* 13 583–594. 10.1007/s00792-009-0261-3 19548063

[B2] Alba K. Kontogiorgos V. (2019). “Seaweed polysaccharides (agar, alginate carrageenan),” in *Encyclopedia of Food Chemistry*, eds Melton L. Shahidi F. Varelis P. (Cambridge, MA: Academic Press), 240–250. 10.1016/b978-0-08-100596-5.21587-4

[B3] Anderson C. T. Kieber J. J. (2020). Dynamic construction, perception, and remodeling of plant cell walls. *Annu. Rev. Plant Biol.* 71 39–69. 10.1146/annurev-arplant-081519-035846 32084323

[B4] Arnosti C. (1995). Measurement of depth- and site-related differences in polysaccharide hydrolysis rates in marine sediments. *Geochimica et Cosmochimica Acta* 59 4247–4257. 10.1016/0016-7037(95)00247-w

[B5] Arnosti C. (1996). A new method for measuring polysaccharide hydrolysis rates in marine environments. *Org. Geochem.* 25 105–115. 10.1016/s0146-6380(96)00112-x

[B6] Arnosti C. (2003). Fluorescent derivatization of polysaccharides and carbohydrate-containing biopolymers for measurement of enzyme activities in complex media. *J. Chromatogr. B Analyt. Technol. Biomed. Life Sci.* 793 181–191. 10.1016/s1570-0232(03)00375-1 12880865

[B7] Aspeborg H. Coutinho P. M. Wang Y. Brumer H. Henrissat B. (2012). Evolution, substrate specificity and subfamily classification of glycoside hydrolase family 5 (GH5). *BMC Evol. Biol.* 12:186. 10.1186/1471-2148-12-186 PMC3526467 22992189

[B8] Barrett K. Lange L. (2019). Peptide-based functional annotation of carbohydrate-active enzymes by conserved unique peptide patterns (CUPP). *Biotechnol. Biofuels* 12:102. 10.1186/s13068-019-1436-5 PMC6489277 31168320

[B9] Bayani J. Squire J. A. (2004). “Fluorescence in situ hybridization (FISH),” in *Current Protocols in Cell Biology*, ed. Bonifacino J. S. (Hoboken, NJ: Wiley). 10.1002/0471143030.cb2204s23 18228455

[B10] Becker S. Tebben J. Coffinet S. Wiltshire K. Iversen M. H. Harder T. (2020). Laminarin is a major molecule in the marine carbon cycle. *Proc. Natl. Acad. Sci. U.S.A.* 117 6599–6607. 10.1073/pnas.1917001117 32170018 PMC7104365

[B11] Berdy B. Spoering A. L. Ling L. L. Epstein S. S. (2017). In situ cultivation of previously uncultivable microorganisms using the ichip. *Nat. Protoc.* 12 2232–2242. 10.1038/nprot.2017.074 29532802

[B12] Börner R. A. (2016). Isolation and cultivation of anaerobes. *Adv. Biochem. Eng. Biotechnol.* 156 35–53. 10.1007/10_2016_1 27028132

[B13] Buck-Wiese H. Fanuel M. Liebeke M. Le Mai Hoang K. Pardo-Vargas A. (2020). Discrimination of β-1,4- and β-1,3-linkages in native oligosaccharides via charge transfer dissociation mass spectrometry. *J. Am. Soc. Mass Spectrom.* 31 1249–1259. 10.1021/jasms.0c00087 32309938

[B14] Burton R. A. Fincher G. B. (2012). Current challenges in cell wall biology in the cereals and grasses [mini review]. *Front. Plant Sci.* 3:130. 10.3389/fpls.2012.00130 PMC3375588 22715340

[B15] Cazy (2021). *Carbohydrate-Active enZYmes.* Available at: http://www.cazy.org. (Accessed January 24, 2021)

[B16] Cherry P. Yadav S. Strain C. R. Allsopp P. J. McSorley E. M. Ross R. P. (2019). Prebiotics from seaweeds: an ocean of opportunity? *Mar. Drugs* 17:327. 10.3390/md17060327 31159359 PMC6627129

[B17] Crouch L. I. Liberato M. V. Urbanowicz P. A. Baslé A. Lamb C. A. Stewart C. J. (2020). Prominent members of the human gut microbiota express endo-acting O-glycanases to initiate mucin breakdown. *Nat. Commun.* 11:4017. 10.1038/s41467-020-17847-5 PMC7419316 32782292

[B18] Cuskin F. Lowe E. C. Temple M. J. Zhu Y. Cameron E. Pudlo N. A. (2015). Human gut bacteroidetes can utilize yeast mannan through a selfish mechanism. *Nature* 517 165–169. 10.1038/nature13995 25567280 PMC4978465

[B19] Dahl W. J. Rivero Mendoza D. Lambert J. M. (2020). Diet, nutrients and the microbiome. *Prog. Mol. Biol. Transl. Sci.* 171 237–263. 10.1016/bs.pmbts.2020.04.006 32475524

[B20] Davani-Davari D. Negahdaripour M. Karimzadeh I. Seifan M. Mohkam M. Masoumi S. J. (2019). Prebiotics: definition, types, sources, mechanisms, and clinical applications. *Foods* 8:92. 10.3390/foods8030092 30857316 PMC6463098

[B21] De Filippo C. Cavalieri D. Di Paola M. Ramazzotti M. Poullet J. B. Massart S. (2010). Impact of diet in shaping gut microbiota revealed by a comparative study in children from Europe and rural Africa. *Proc. Natl. Acad. Sci. U.S.A.* 107 14691–14696. 10.1073/pnas.1005963107 20679230 PMC2930426

[B22] Doco T. Williams P. Meudec E. Cheynier V. Sommerer N. (2015). Complex carbohydrates of red wine: characterization of the extreme diversity of neutral oligosaccharides by ESI-MS. *J. Agric. Food Chem.* 63 671–682. 10.1021/jf504795g 25530549

[B23] Duarte M. L. Ferreira M. C. Marvao M. R. Rocha J. (2002). An optimised method to determine the degree of acetylation of chitin and chitosan by FTIR spectroscopy. *Int. J. Biol. Macromol.* 31 1–8. 10.1016/s0141-8130(02)00039-9 12559421

[B24] El Kaoutari A. Armougom F. Gordon J. I. Raoult D. Henrissat B. (2013). The abundance and variety of carbohydrate-active enzymes in the human gut microbiota. *Nat. Rev. Microbiol.* 11 497–504. 10.1038/nrmicro3050 23748339

[B25] Fernandez X. Tornberg E. V. A. (1991). A review of the causes of variation in muscle glycogen content and ultimate pH in pigs. *J. Muscle Foods* 2 209–235. 10.1111/j.1745-4573.1991.tb00454.x

[B26] Flamm G. Glinsmann W. Kritchevsky D. Prosky L. Roberfroid M. (2001). Inulin and oligofructose as dietary fiber: a review of the evidence. *Crit. Rev. Food Sci. Nutr.* 41 353–362. 10.1080/20014091091841 11497328

[B27] Forján E. Navarro F. Cuaresma M. Vaquero I. Ruíz-Domínguez M. C. Gojkovic Ž (2014). Microalgae: fast-growth sustainable green factories. *Crit. Rev. Environ. Sci. Technol.* 45 1705–1755. 10.1080/10643389.2014.966426

[B28] Gentile C. L. Weir T. L. (2018). The gut microbiota at the intersection of diet and human health. *Science* 362 776–780. 10.1126/science.aau5812 30442802 PMC13264711

[B29] Gibson G. R. Hutkins R. Sanders M. E. Prescott S. L. Reimer R. A. Salminen S. J. (2017). Expert consensus document: the international scientific association for probiotics and prebiotics (ISAPP) consensus statement on the definition and scope of prebiotics. *Nat. Rev. Gastroenterol. Hepatol.* 14 491–502. 10.1038/nrgastro.2017.75 28611480

[B30] Glabe C. G. Harty P. K. Rosen S. D. (1983). Preparation and properties of fluorescent polysaccharides. *Anal. Biochem.* 130 287–294. 10.1016/0003-2697(83)90590-0 6869815

[B31] Glenn W. S. Stone S. E. Ho S. H. Sweredoski M. J. Moradian A. Hess S. (2017). Bioorthogonal noncanonical amino acid tagging (BONCAT) enables time-resolved analysis of protein synthesis in native plant tissue. *Plant Physiol.* 173 1543–1553. 10.1104/pp.16.01762 28104718 PMC5338676

[B32] Gnoth M. J. Kunz C. Kinne-Saffran E. Rudloff S. (2000). Human milk oligosaccharides are minimally digested in vitro. *J. Nutr.* 130 3014–3020. 10.1093/jn/130.12.3014 11110861

[B33] Gow N. A. R. Latge J.-P. Munro C. A. (2017). The fungal cell wall: structure, biosynthesis, and function. *Microbiol. Spectr.* 5, 1–25. 10.1128/microbiolspec.funk-0035-2016 PMC11687499 28513415

[B34] Grondin J. M. Tamura K. Déjean G. Abbott D. W. Brumer H. (2017). Polysaccharide utilization loci: fueling microbial communities. *J. Bacteriol.* 199:e000860-e16. 10.1128/JB.00860-16 PMC5512228 28138099

[B35] Hatzenpichler R. Krukenberg V. Spietz R. L. Jay Z. J. (2020). Next-generation physiology approaches to study microbiome function at single cell level. *Nat. Rev. Microbiol.* 18 241–256. 10.1038/s41579-020-0323-1 32055027 PMC7133793

[B36] Hehemann J. H. Reintjes G. Klassen L. Smith A. D. Ndeh D. Arnosti C. (2019). Single cell fluorescence imaging of glycan uptake by intestinal bacteria. *ISME J.* 13 1883–1889. 10.1038/s41396-019-0406-z 30936421 PMC6776043

[B37] Hehemann J.-H. Kelly A. G. Pudlo N. A. Martens E. C. Boraston A. B. (2012). Bacteria of the human gut microbiome catabolize red seaweed glycans with carbohydrate-active enzyme updates from extrinsic microbes. *Proc. Natl. Acad. Sci. U.S.A.* 109 19786–19791. 10.1073/pnas.1211002109 23150581 PMC3511707

[B38] Helbert W. Poulet L. Drouillard S. Mathieu S. Loiodice M. Couturier M. (2019). Discovery of novel carbohydrate-active enzymes through the rational exploration of the protein sequences space. *Proc. Natl. Acad. Sci. U.S.A.* 116 6063–6068. 10.1073/pnas.1815791116 30850540 PMC6442616

[B39] Honda K. Littman D. R. (2016). The microbiota in adaptive immune homeostasis and disease. *Nature* 535 75–84. 10.1038/nature18848 27383982

[B40] Hong V. Steinmetz N. F. Manchester M. Finn M. G. (2010). Labeling live cells by copper-catalyzed alkyne–azide click chemistry. *Bioconjug. Chem.* 21 1912–1916. 10.1021/bc100272z 20886827 PMC3014321

[B41] Hsieh Y. S. Y. Harris P. J. (2019). Xylans of red and green algae: what is known about their structures and how they are synthesised? *Polymers* 11:354. 10.3390/polym11020354 30960338 PMC6419167

[B42] Jaeger D. Ndi C. P. Crocoll C. Simpson B. S. Khakimov B. Guzman-Genuino R. M. (2017). Isolation and structural characterization of echinocystic acid triterpenoid saponins from the australian medicinal and food plant acacia ligulata. *J. Nat. Prod.* 80 2692–2698. 10.1021/acs.jnatprod.7b00437 28976773

[B43] Jha A. R. Davenport E. R. Gautam Y. Bhandari D. Tandukar S. Ng K. M. (2018). Gut microbiome transition across a lifestyle gradient in himalaya. *PLoS Biol.* 16:e2005396. 10.1371/journal.pbio.2005396 30439937 PMC6237292

[B44] Jones D. R. Thomas D. Alger N. Ghavidel A. Inglis G. D. Abbott D. W. (2018). SACCHARIS: an automated pipeline to streamline discovery of carbohydrate active enzyme activities within polyspecific families and de novo sequence datasets. *Biotechnol. Biofuels* 11:27. 10.1186/s13068-018-1027-x PMC5798181 29441125

[B45] Kanyer A. J. Bornhorst G. M. Marco M. L. Bamforth C. W. (2017). Is beer a source of prebiotics? *J. Inst. Brew.* 123 361–365. 10.1002/jib.439

[B46] Kidgell J. T. Magnusson M. de Nys R. Glasson C. R. K. (2019). Ulvan: a systematic review of extraction, composition and function. *Algal Res.* 39:101422. 10.1016/j.algal.2019.101422

[B47] Klassen L. Reintjes G. Tingley J. P. Jones D. R. Hehemann J. H. Smith A. D. (2021). Quantifying fluorescent glycan uptake to elucidate strain-level variability in foraging behaviors of rumen bacteria. *Microbiome* 9:23. 10.1186/s40168-020-00975-x PMC7825182 33482928

[B48] Kolb H. C. Finn M. G. Sharpless K. B. (2001). Click chemistry: diverse chemical function from a few good reactions. *Angew. Chem. Int. Ed. Engl.* 40 2004–2021. 10.1002/1521-3773(20010601)40:11<2004::aid-anie2004>3.0.co;2-5 11433435

[B49] Komatsu T. Shoji N. Saito K. Suzuki K. (2014). Effects of genetic and environmental factors on muscle glycogen content in Japanese black cattle. *Anim. Sci. J.* 85 793–798. 10.1111/asj.12201 24716455 PMC4271676

[B50] Krajmalnik-Brown R. Ilhan Z. E. Kang D. W. DiBaise J. K. (2012). Effects of gut microbes on nutrient absorption and energy regulation. *Nutr. Clin. Pract.* 27 201–214. 10.1177/0884533611436116 22367888 PMC3601187

[B51] La Fata G. Weber P. Mohajeri M. H. (2018). Probiotics and the gut immune system: indirect regulation. *Probiotics Antimicrob. Proteins* 10 11–21. 10.1007/s12602-017-9322-6 28861741 PMC5801397

[B52] Lee K. S. Palatinszky M. Pereira F. C. Nguyen J. Fernandez V. I. Mueller A. J. (2019). An automated raman-based platform for the sorting of live cells by functional properties. *Nat. Microbiol.* 4 1035–1048. 10.1038/s41564-019-0394-9 30886359

[B53] Letunic I. Bork P. (2019). Interactive tree of life (iTOL) v4: recent updates and new developments. *Nucleic Acids Res.* 47 W256–W259. 30931475 10.1093/nar/gkz239 PMC6602468

[B54] Li J. Wang D. Xing X. Cheng T. R. Liang P. H. Bulone V. (2019). Structural analysis and biological activity of cell wall polysaccharides extracted from Panax ginseng marc. *Int. J. Biol. Macromol.* 135 29–37. 10.1016/j.ijbiomac.2019.05.077 31121231

[B55] Li T. Wu T. D. Mazéas L. Toffin L. Guerquin-Kern J. L. Leblon G. (2008). Simultaneous analysis of microbial identity and function using NanoSIMS. *Environ. Microbiol.* 10 580–588. 10.1111/j.1462-2920.2007.01478.x 18028417 PMC2253709

[B56] Li Z. Quan G. Jiang X. Yang Y. Ding X. Zhang D. (2018). Effects of metabolites derived from gut microbiota and hosts on pathogens. *Front. Cell Infect Microbiol.* 8:314. 10.3389/fcimb.2018.00314 PMC6152485 30276161

[B57] Little A. Lahnstein J. Jeffery D. W. Khor S. F. Schwerdt J. G. Shirley N. J. (2019). A NOVEL (1,4)-beta-linked glucoxylan is synthesized by members of the Cellulose synthase-like f gene family in land plants. *ACS Cent. Sci.* 5 73–84. 10.1021/acscentsci.8b00568 30693327 PMC6346400

[B58] Lobb B. Tremblay B. J. Moreno-Hagelsieb G. Doxey A. C. (2020). An assessment of genome annotation coverage across the bacterial tree of life. *Microb. Genom* 6:e000341. 10.1099/mgen.0.000341 PMC7200070 32124724

[B59] LoCascio R. G. Ninonuevo M. R. Freeman S. L. Sela D. A. Grimm R. Lebrilla C. B. (2007). Glycoprofiling of bifidobacterial consumption of human milk oligosaccharides demonstrates strain specific, preferential consumption of small chain glycans secreted in early human lactation. *J. Agric. Food Chem.* 55 8914–8919. 10.1021/jf0710480 17915960

[B60] Lombard V. Golaconda Ramulu H. Drula E. Coutinho P. M. Henrissat B. (2014). The carbohydrate-active enzymes database (CAZy) in 2013. *Nucleic Acids Res.* 42 D490–D495. 24270786 10.1093/nar/gkt1178 PMC3965031

[B61] Mager L. F. Burkhard R. Pett N. Cooke N. C. A. Brown K. Ramay H. (2020). Microbiome-derived inosine modulates response to checkpoint inhibitor immunotherapy. *Science* 369 1481–1489. 10.1126/science.abc3421 32792462

[B62] Marcobal A. Barboza M. Sonnenburg E. D. Pudlo N. Martens E. C. Desai P. (2011). Bacteroides in the infant gut consume milk oligosaccharides via mucus-utilization pathways. *Cell Host Microbe* 10 507–514. 10.1016/j.chom.2011.10.007 22036470 PMC3227561

[B63] Marcotuli I. Colasuonno P. Hsieh Y. S. Y. Fincher G. B. Gadaleta A. (2020). Non-starch polysaccharides in durum wheat: a review. *Int. J. Mol. Sci.* 21:2933. 10.3390/ijms21082933 32331292 PMC7215680

[B64] Mellor D. B. Stringer P. A. Mountney G. J. (1958). The influence of glycogen on the tenderness of broiler meat. *Poult. Sci.* 37 1028–1034. 10.3382/ps.0371028

[B65] Mewis K. Lenfant N. Lombard V. Henrissat B. (2016). Dividing the large Glycoside hydrolase family 43 into Subfamilies: a motivation for detailed enzyme characterization. *Appl. Environ. Microbiol.* 82 1686–1692. 10.1128/aem.03453-15 26729713 PMC4784025

[B66] Mirhosseini H. Amid B. T. (2012). A review study on chemical composition and molecular structure of newly plant gum exudates and seed gums. *Food Res. Int.* 46 387–398. 10.1016/j.foodres.2011.11.017

[B67] Monteagudo-Mera A. Rastall R. A. Gibson G. R. Charalampopoulos D. Chatzifragkou A. (2019). Adhesion mechanisms mediated by probiotics and prebiotics and their potential impact on human health. *Appl. Microbiol. Biotechnol.* 103 6463–6472. 10.1007/s00253-019-09978-7 31267231 PMC6667406

[B68] Morales V. Sanz M. L. Olano A. Corzo N. (2006). Rapid Separation on activated charcoal of high oligosaccharides in honey. *Chromatographia* 64:53.

[B69] Ndeh D. Rogowski A. Cartmell A. Luis A. S. Baslé A. Gray J. (2017). Complex pectin metabolism by gut bacteria reveals novel catalytic functions. *Nature* 544 65–70. 10.1038/nature21725 28329766 PMC5388186

[B70] Neelamegham S. Aoki-Kinoshita K. Bolton E. Frank M. Lisacek F. Lütteke T. (2019). Updates to the symbol nomenclature for glycans guidelines. *Glycobiology* 29 620–624. 10.1093/glycob/cwz045 31184695 PMC7335484

[B71] Oliveira D. L. Wilbey R. A. Grandison A. S. Roseiro L. B. (2015). Milk oligosaccharides: a review. *Int. J. Dairy Technol.* 68 305–321.

[B72] Osemwegie O. O. Adetunji C. O. Ayeni E. A. Adejobi O. I. Arise R. O. Nwonuma C. O. (2020). Exopolysaccharides from bacteria and fungi: current status and perspectives in Africa. *Heliyon* 6:e04205. 10.1016/j.heliyon.2020.e04205 32577572 PMC7303563

[B73] Ozkul C. Yalinay M. Karakan T. (2020). Structural changes in gut microbiome after Ramadan fasting: a pilot study. *Benef. Microbes* 11 227–233. 10.3920/bm2019.0039 32073296

[B74] Padayachee A. Day L. Howell K. Gidley M. J. (2017). Complexity and health functionality of plant cell wall fibers from fruits and vegetables. *Crit. Rev. Food Sci. Nutr.* 57 59–81. 10.1080/10408398.2013.850652 25830345

[B75] Pandya U. Dhuldhaj U. Sahay N. S. (2019). Bioactive mushroom polysaccharides as antitumor: an overview. *Nat. Prod. Res.* 33 2668–2680. 10.1080/14786419.2018.1466129 29726720

[B76] Pangestuti R. Arifin Z. (2018). Medicinal and health benefit effects of functional sea cucumbers. *J. Tradit. Complement Med.* 8 341–351. 10.1016/j.jtcme.2017.06.007 29992105 PMC6035309

[B77] Pittman Q. J. (2020). A gut feeling about the ketogenic diet in epilepsy. *Epilepsy Res.* 166:106409. 10.1016/j.eplepsyres.2020.106409 32673970

[B78] Plaza-Díaz J. Ruiz-Ojeda F. J. Vilchez-Padial L. M. Gil A. (2017). Evidence of the anti-inflammatory effects of probiotics and synbiotics in intestinal chronic diseases. *Nutrients* 9:555. 10.3390/nu9060555 28555037 PMC5490534

[B79] Pluvinage B. Grondin J. M. Amundsen C. Klassen L. Moote P. E. Xiao Y. (2018). Molecular basis of an agarose metabolic pathway acquired by a human intestinal symbiont. *Nat. Commun.* 9:1043. 10.1038/s41467-018-03366-x PMC5849685 29535379

[B80] Praveen M. A. Parvathy K. R. K. Balasubramanian P. Jayabalan R. (2019). An overview of extraction and purification techniques of seaweed dietary fibers for immunomodulation on gut microbiota. *Trends Food Sci. Technol.* 92 46–64. 10.1016/j.tifs.2019.08.011

[B81] Reichart N. J. Jay Z. J. Krukenberg V. Parker A. E. Spietz R. L. Hatzenpichler R. (2020). Activity-based cell sorting reveals responses of uncultured archaea and bacteria to substrate amendment. *ISME J.* 14 2851–2861. 10.1038/s41396-020-00749-1 32887944 PMC7784905

[B82] Reintjes G. Arnosti C. Fuchs B. M. Amann R. (2017). An alternative polysaccharide uptake mechanism of marine bacteria [original article]. *ISME J.* 11 1640–1650. 10.1038/ismej.2017.26 28323277 PMC5520146

[B83] Roberts A. W. Lahnstein J. Hsieh Y. S. Y. Xing X. Yap K. Chaves A. M. (2018). Functional characterization of a glycosyltransferase from the moss physcomitrella patens involved in the biosynthesis of a novel cell wall arabinoglucan. *Plant Cell* 30 1293–1308. 10.1105/tpc.18.00082 29674386 PMC6048786

[B84] Ropartz D. Li P. Fanuel M. Giuliani A. Rogniaux H. Jackson G. P. (2016). Charge transfer dissociation of complex oligosaccharides: comparison with collision-induced dissociation and extreme ultraviolet dissociative photoionization. *J. Am. Soc. Mass Spectrom.* 27 1614–1619. 10.1007/s13361-016-1453-6 27582116

[B85] Ropartz D. Li P. Jackson G. P. Rogniaux H. (2017). Negative polarity helium charge transfer dissociation tandem mass spectrometry: radical-initiated fragmentation of complex polysulfated anions. *Anal. Chem.* 89 3824–3828. 10.1021/acs.analchem.7b00473 28300396

[B86] Saffer A. M. (2018). Expanding roles for pectins in plant development. *J. Integr. Plant Biol.* 60 910–923. 10.1111/jipb.12662 29727062

[B87] Saha D. Bhattacharya S. (2010). Hydrocolloids as thickening and gelling agents in food: a critical review. *J. Food Sci. Technol.* 47 587–597. 10.1007/s13197-010-0162-6 23572691 PMC3551143

[B88] Salzberg S. L. (2019). Next-generation genome annotation: we still struggle to get it right. *Genome Biol.* 20:92. 10.1186/s13059-019-1715-2 PMC6521345 31097009

[B89] Sato K. Nagai N. Yamamoto T. Mitamura K. Taga A. (2019). Identification of a novel oligosaccharide in maple syrup as a potential alternative saccharide for diabetes mellitus patients. *Int. J. Mol. Sci.* 20:5041. 10.3390/ijms20205041 31614552 PMC6834145

[B90] Schmid J. Sieber V. Rehm B. (2015). Bacterial exopolysaccharides: biosynthesis pathways and engineering strategies [review]. *Front. Microbiol.* 6:496. 10.3389/fmicb.2015.00496 PMC4443731 26074894

[B91] Scott K. P. Grimaldi R. Cunningham M. Sarbini S. R. Wijeyesekera A. Tang M. L. K. (2020). Developments in understanding and applying prebiotics in research and practice—an ISAPP conference paper. *J. Appl. Microbiol.* 128 934–949. 10.1111/jam.14424 31446668

[B92] Scott K. P. Martin J. C. Duncan S. H. Flint H. J. (2014). Prebiotic stimulation of human colonic butyrate-producing bacteria and bifidobacteria, in vitro. *FEMS Microbiol. Ecol.* 87 30–40. 10.1111/1574-6941.12186 23909466

[B93] Shaffer M. Borton M. A. McGivern B. B. Zayed A. A. La Rosa S. L. Solden L. M. (2020). DRAM for distilling microbial metabolism to automate the curation of microbiome function. *Nucleic Acids Res.* 48 8883–8900. 10.1093/nar/gkaa621 32766782 PMC7498326

[B94] Sivam A. S. Sun-Waterhouse D. Quek S. Perera C. O. (2010). Properties of bread dough with added fiber polysaccharides and phenolic antioxidants: a review. *J. Food Sci.* 75 R163–R174. 21535512 10.1111/j.1750-3841.2010.01815.x PMC3032915

[B95] Smits S. A. Leach J. Sonnenburg E. D. Gonzalez C. G. Lichtman J. S. Reid G. (2017). Seasonal cycling in the gut microbiome of the hadza hunter-gatherers of Tanzania. *Science* 357 802–806. 10.1126/science.aan4834 28839072 PMC5891123

[B96] Sonnenburg E. D. Sonnenburg J. L. (2019). The ancestral and industrialized gut microbiota and implications for human health. *Nat. Rev. Microbiol.* 17 383–390. 10.1038/s41579-019-0191-8 31089293

[B97] St John F. J. González J. M. Pozharski E. (2010). Consolidation of glycosyl hydrolase family 30: a dual domain 4/7 hydrolase family consisting of two structurally distinct groups. *FEBS Lett.* 584 4435–4441. 10.1016/j.febslet.2010.09.051 20932833

[B98] Stam M. R. Danchin E. G. Rancurel C. Coutinho P. M. Henrissat B. (2006). Dividing the large glycoside hydrolase family 13 into subfamilies: towards improved functional annotations of alpha-amylase-related proteins. *Protein Eng. Des. Sel.* 19 555–562. 10.1093/protein/gzl044 17085431

[B99] Stewart R. D. Auffret M. D. Roehe R. Watson M. (2018). Open prediction of polysaccharide utilisation loci (PUL) in 5414 public bacteroidetes genomes using PULpy. *bioRxiv [Preprint]* 10.1101/421024

[B100] Tamura K. Hemsworth G. R. Déjean G. Rogers T. E. Pudlo N. A. Urs K. (2017). Molecular mechanism by which prominent human gut bacteroidetes utilize mixed-linkage beta-glucans, major health-promoting cereal polysaccharides. *Cell Rep.* 21 417–430. 10.1016/j.celrep.2017.09.049 29020628 PMC5656003

[B101] Tancula E. Feldhaus M. J. Bedzyk L. A. Salyers A. A. (1992). Location and characterization of genes involved in binding of starch to the surface of *Bacteroides thetaiotaomicron*. *J. Bacteriol.* 174 5609–5616. 10.1128/jb.174.17.5609-5616.1992 1512196 PMC206506

[B102] Tao J. McCourt C. Sultana H. Nelson C. Driver J. Hackmann T. J. (2019). Use of a fluorescent analog of glucose (2-NBDG) to identify uncultured rumen bacteria that take up glucose. *Appl. Environ. Microbiol.* 85:e03018-18. 10.1128/AEM.03018-18 PMC6585498 30709823

[B103] Thinh P. D. Ly B. M. Usoltseva R. V. Shevchenko N. M. Rasin A. B. Anastyuk S. D. (2018). A novel sulfated fucan from vietnamese sea cucumber stichopus variegatus: isolation, structure and anticancer activity in vitro. *Int. J. Biol. Macromol.* 117 1101–1109. 10.1016/j.ijbiomac.2018.06.017 29885396

[B104] Tingley J. P. Low K. E. Xing X. Abbott D. W. (2021). Combined whole cell wall analysis and streamlined in silico carbohydrate-active enzyme discovery to improve biocatalytic conversion of agricultural crop residues. *Biotechnol. Biofuels* 14:16. 10.1186/s13068-020-01869-8 PMC7797155 33422151

[B105] Usov A. I. (2011). “Chapter 4 - polysaccharides of the red algae,” in *Advances in Carbohydrate Chemistry and Biochemistry*, Vol. 65 ed. Horton D. (Cambridgr, MA: Academic Press), 115–217. 10.1016/b978-0-12-385520-6.00004-2 21763512

[B106] Ustyuzhanina N. E. Bilan M. I. Dmitrenok A. S. Nifantiev N. E. Usov A. I. (2018). Fucosylated chondroitin sulfates from the sea cucumbers *Holothuria tubulosa* and *Holothuria stellati*. *Carbohydr. Polym.* 200 1–5. 10.1016/j.carbpol.2018.07.035 30177144

[B107] Valdes A. M. Walter J. Segal E. Spector T. D. (2018). Role of the gut microbiota in nutrition and health. *Bmj* 361:k2179. 10.1136/bmj.k2179 29899036 PMC6000740

[B108] Varki A. Cummings R. D. Aebi M. Packer N. H. Seeberger P. H. Esko J. D. (2015). Symbol nomenclature for graphical representations of glycans. *Glycobiology* 25 1323–1324. 26543186 10.1093/glycob/cwv091 PMC4643639

[B109] Viborg A. H. Terrapon N. Lombard V. Michel G. Czjzek M. Henrissat B. (2019). A subfamily roadmap of the evolutionarily diverse glycoside hydrolase family 16 (GH16). *J. Biol. Chem.* 294 15973–15986. 10.1074/jbc.ra119.010619 31501245 PMC6827312

[B110] Villarruel-López A. Ascencio F. Nuño K. (2017). Microalgae, a potential natural functional food source – a review. *Pol. J. Food Nutr. Sci.* 67 251–263. 10.1515/pjfns-2017-0017

[B111] Vinogradov E. Petersen B. O. Duus J. Wasser S. (2004). The structure of the glucuronoxylomannan produced by culinary-medicinal yellow brain mushroom (*Tremella mesenterica* Ritz.:Fr., Heterobasidiomycetes) grown as one cell biomass in submerged culture. *Carbohydr. Res.* 339 1483–1489. 10.1016/j.carres.2004.04.001 15178391

[B112] Wagner M. (2009). Single-cell ecophysiology of microbes as revealed by raman microspectroscopy or secondary ion mass spectrometry imaging. *Annu. Rev. Microbiol.* 63 411–429. 10.1146/annurev.micro.091208.073233 19514853

[B113] Wang W. Yang Q. Du Y. Zhou X. Du X. Wu Q. (2020). Metabolic labeling of peptidoglycan with Nir-II dye enables in vivo imaging of gut microbiota. *Angew. Chem. Int. Ed Engl.* 59 2628–2633. 10.1002/anie.201910555 31793153

[B114] Wood J. A. Tan H. T. Collins H. M. Yap K. Khor S. F. Lim W. L. (2018). Genetic and environmental factors contribute to variation in cell wall composition in mature desi chickpea (*Cicer arietinum* L.) cotyledons. *Plant Cell Environ.* 41 2195–2208. 29532951 10.1111/pce.13196

[B115] Wu Y. J. Wei Z. X. Zhang F. M. Linhardt R. J. Sun P. L. Zhang A. Q. (2019). Structure, bioactivities and applications of the polysaccharides from *Tremella fuciformis* mushroom: a review. *Int. J. Biol. Macromol.* 121 1005–1010. 10.1016/j.ijbiomac.2018.10.117 30342120

[B116] Xing X. H. Zhang Z. M. Hu X. Z. Wu R. Q. Xu C. (2009). Antidiabetic effects of Artemisia sphaerocephala Krasch. gum, a novel food additive in China, on streptozotocin-induced type 2 diabetic rats. *J. Ethnopharmacol.* 125 410–416. 10.1016/j.jep.2009.07.021 19635546

[B117] Xing X. Cui S. W. Nie S. Phillips G. O. Douglas Goff H. Wang Q. (2013). A review of isolation process, structural characteristics, and bioactivities of water-soluble polysaccharides from dendrobium plants. *Bioactive Carbohydrates Dietary Fibre* 1 131–147. 10.1016/j.bcdf.2013.04.001

[B118] Xing X. Cui S. W. Nie S. Phillips G. O. Goff H. D. Wang Q. (2014). Study on dendrobium officinale O-acetyl-glucomannan (dendronan^®^): part i. extraction, purification, and partial structural characterization. *Bioactive Carbohydrates Dietary Fibre* 4 74–83. 10.1016/j.bcdf.2014.06.004

[B119] Xing X. Cui S. W. Nie S. Phillips G. O. Goff H. D. Wang Q. (2015a). Study on Dendrobium officinale O-acetyl-glucomannan (Dendronan^®^): part II. fine structures of O-acetylated residues. *Carbohydr. Polm.* 117 422–433. 10.1016/j.carbpol.2014.08.121 25498655

[B120] Xing X. Cui S. W. Nie S. Phillips G. O. Goff H. D. Wang Q. (2015b). Study on Dendrobium officinale O-acetyl-glucomannan (Dendronan^®^): part V. fractionation and structural heterogeneity of different fractions. *Bioactive Carbohydrates and Dietary Fibre* 5 106–115. 10.1016/j.bcdf.2014.12.005

[B121] Xing X. Hsieh Y. S. Y. Yap K. Ang M. E. Lahnstein J. Tucker M. R. (2017). Isolation and structural elucidation by 2D NMR of planteose, a major oligosaccharide in the mucilage of chia (*Salvia hispanica* L.) seeds. *Carbohydr. Polym.* 175 231–240. 10.1016/j.carbpol.2017.07.059 28917861

[B122] Xiong Q. Song Z. Hu W. Liang J. Jing Y. He L. (2020). Methods of extraction, separation, purification, structural characterization for polysaccharides from aquatic animals and their major pharmacological activities. *Crit. Rev. Food Sci. Nutr.* 60 48–63. 10.1080/10408398.2018.1512472 30285473

[B123] Yan H. Yalagala R. S. Yan F. (2015). Fluorescently labelled glycans and their applications. *Glycoconj. J.* 32 559–574. 10.1007/s10719-015-9611-9 26239924

[B124] Yu L. Yakubov G. E. Zeng W. Xing X. Stenson J. Bulone V. (2017). Multi-layer mucilage of *Plantago ovata* seeds: rheological differences arise from variations in arabinoxylan side chains. *Carbohydr. Polym.* 165 132–141. 10.1016/j.carbpol.2017.02.038 28363533

[B125] Zhang H. Yohe T. Huang L. Entwistle S. Wu P. Yang Z. (2018). dbCAN2: a meta server for automated carbohydrate-active enzyme annotation. *Nucleic Acids Res.* 46 W95–W101. 29771380 10.1093/nar/gky418 PMC6031026

[B126] Zhang X. Zhang Y. (2013). Applications of azide-based bioorthogonal click chemistry in glycobiology. *Molecules* 18 7145–7159. 10.3390/molecules18067145 23783454 PMC6269833

[B127] Zhu Y. Suits M. D. Thompson A. J. Chavan S. Dinev Z. Dumon C. (2010). Mechanistic insights into a Ca2+-dependent family of alpha-mannosidases in a human gut symbiont. *Nat. Chem. Biol.* 6 125–132. 10.1038/nchembio.278 20081828 PMC3942423

