# Supplementary material for: Approaches to Investigate Selective Dietary Polysaccharide Utilization by Human Gut Microbiota at a Functional Level
Source: Front Microbiol. 2021 Feb 19;12:632684. doi: 10.3389/fmicb.2021.632684 (PMC7933471; doi:10.3389/fmicb.2021.632684)
Supplement: Supplementary file 1 [file Data_Sheet_1.pdf]

Supplementary Material

Supplementary Table 1. Structural diversity of complex carbohydrates from various biological sources of foods

Sources	Food samples	Carbohydrate examples	References
Higher plants	Cereal crops, legumes, fruits, vegetables, roots, tubers; Isolated mucilage polysaccharides as food gum; maple syrup	Cellulose, β-(1,3;1,4)-glucan, callose, glucomannan, galactomannan, heteroxylan, xyloglucan, arabinoxylan, arabinan, rhamnogalacturonan, arabinogalactan, starch, fructan.	(Anderson and Kieber 2020, Burton and Fincher 2012, Marcotuli et al. 2020, Mirhosseini and Amid 2012, Padayachee et al. 2017, Saffer 2018, Sato et al. 2019)
Fungi	Edible mushrooms	Chitin, β-(1,3)-glucan, β-(1,3;1,6)-glucan, β-(1,6)-glucan, glycogen-like α-glucan, mannoprotein, glucuronoxylomannan.	(Cuskin et al. 2015, Gow et al. 2017, Pandya et al. 2019, Vinogradov et al. 2004, Wu et al. 2019)
Microalgae	Isolated polysaccharide as food gum	Sulfated exopolysaccharides, β-(1,3)-glucan, starch, laminarin,	(Forján et al. 2014, Villarruel-López et al. 2017)
Bacteria	Exopolysaccharide as food gum	Xanthan gum, gellan gum, , alginate, cellulose, curdlan, dextran, hyaluronic acid, levan.	(Osemwegie et al. 2020, Schmid et al. 2015, Wu et al. 2009)
Seaweed	Edible red, brown, and green seaweeds	Alginate, agarose, porphyran, carrageenan, fucoidan, ulvan, cellulose, β-1,3-xylan, β-1,4-xylan, β-(1,3;1,4)-xylan, β-1,4-mannan, xylomannan, furcellaran, laminarin.	(Alba and Kontogiorgos 2019, Becker et al. 2020, Cherry et al. 2019, Hsieh and Harris 2019, Kidgell et al. 2019, Kinnaert et al. 2017, Praveen et al. 2019, Tanna and Mishra 2019, Usov 2011)

Marine crustacean shell	Chitosan produced from chitin in crab and shrimp shell	Chitin, chitosan	(Hamed et al. 2016, Manigandan et al. 2018, Suryawanshi et al. 2019)
Marine animal	Sea cucumber	Sulfated fucan, fucosylated chondroitin sulfates	(Pangestuti and Arifin 2018, Thinh et al. 2018, Ustyuzhanina et al. 2018, Xiong et al. 2020)
Animal (meat)	beef, pork, poultry muscle meat; liver of duck or goose for *foie gras* production	Glycogen	(Bonnefont et al. 2019, Fernandez and Tornberg 1991, Komatsu et al. 2014, Mellor et al. 1958)
Animal secretion	milk, honey, human breast milk	Fructo-oligosaccharides, Galacto-oligosaccharides, human milk oligosaccharides (*e.g.* 3'-Sialyllactose, Fucosyllactose, and Lacto-N-Neohexaose), other complex oligosaccharides	(Kanyer et al. 2017, Morales et al. 2006, Oliveira et al. 2015)
Complex sources	Complex processed food (*e.g.* bread, noodles, cola, wine, beer)	Mixture of complex carbohydrates, such as starch and rhamnogalacturonan II	(Doco et al. 2015, Kanyer, Bornhorst, Marco and Bamforth 2017, Saha and Bhattacharya 2010, Sivam et al. 2010)

References

Alba K, Kontogiorgos V. 2019. Seaweed Polysaccharides (Agar, Alginate Carrageenan). In: Encyclopedia of Food Chemistry. Oxford: Academic Press. p. 240-250.

Anderson CT, Kieber JJ. 2020. Dynamic Construction, Perception, and Remodeling of Plant Cell Walls. Annu Rev Plant Biol. Apr 29;71:39-69. Epub 2020/02/23.

Becker S, Tebben J, Coffinet S, Wiltshire K, Iversen MH, Harder T, Hinrichs KU, Hehemann JH. 2020. Laminarin is a major molecule in the marine carbon cycle. Proc Natl Acad Sci U S A. Mar 24;117:6599-6607. Epub 2020/03/15.

Bonnefont CMD, Molette C, Lavigne F, Manse H, Bravo C, Lo B, Remignon H, Arroyo J, Bouillier-Oudot M. 2019. Evolution of liver fattening and foie gras technological yield during the overfeeding period in mule duck. Poult Sci. Nov 1;98:5724-5733. Epub 2019/07/28.

Burton RA, Fincher GB. 2012. Current challenges in cell wall biology in the cereals and grasses. Front Plant Sci. 2012-June-15;3:130. Epub 2012/06/21.

Cherry P, Yadav S, Strain CR, Allsopp PJ, McSorley EM, Ross RP, Stanton C. 2019. Prebiotics from Seaweeds: An Ocean of Opportunity? Mar Drugs. Jun 1;17. Epub 2019/06/05.

Cuskin F, Lowe EC, Temple MJ, Zhu Y, Cameron E, Pudlo NA, Porter NT, Urs K, Thompson AJ, Cartmell A, et al. 2015. Human gut Bacteroidetes can utilize yeast mannan through a selfish mechanism. Nature. Jan;517:165-169.

Doco T, Williams P, Meudec E, Cheynier V, Sommerer N. 2015. Complex carbohydrates of red wine: characterization of the extreme diversity of neutral oligosaccharides by ESI-MS. J Agric Food Chem. Jan 21;63:671-682. Epub 2014/12/23.

Fernandez X, Tornberg EVA. 1991. A review of the causes of variation in muscle glycogen content and ultimate pH in pigs. Journal of Muscle Foods.2:209-235.

Forján E, Navarro F, Cuaresma M, Vaquero I, Ruíz-Domínguez MC, Gojkovic Ž, Vázquez M, Márquez M, Mogedas B, Bermejo E, et al. 2014. Microalgae: Fast-Growth Sustainable Green Factories. Critical Reviews in Environmental Science and Technology. 2015/08/18;45:1705-1755.

Gow NAR, Latge J-P, Munro CA. 2017. The fungal cell wall: Structure, biosynthesis, and function. In: The Fungal Kingdom. American Society of Microbiology.

Hamed I, Özogul F, Regenstein JM. 2016. Industrial applications of crustacean by-products (chitin, chitosan, and chitooligosaccharides): A review. Trends in Food Science & Technology. 2016/02/01/;48:40-50.

Hsieh YSY, Harris PJ. 2019. Xylans of Red and Green Algae: What Is Known about Their Structures and How They Are Synthesised? Polymers (Basel). Feb 18;11. Epub 2019/04/10.

Kanyer AJ, Bornhorst GM, Marco ML, Bamforth CW. 2017. Is beer a source of prebiotics? Journal of the Institute of Brewing.123:361-365.

Kidgell JT, Magnusson M, de Nys R, Glasson CRK. 2019. Ulvan: A systematic review of extraction, composition and function. Algal Research. 2019/05/01/;39:101422.

Kinnaert C, Daugaard M, Nami F, Clausen MH. 2017. Chemical Synthesis of Oligosaccharides Related to the Cell Walls of Plants and Algae. Chem Rev. Sep 13;117:11337-11405. Epub 2017/08/10.

Komatsu T, Shoji N, Saito K, Suzuki K. 2014. Effects of genetic and environmental factors on muscle glycogen content in Japanese Black cattle. Anim Sci J. Aug;85:793-798. Epub 2014/04/11.

Manigandan V, Karthik R, Ramachandran S, Rajagopal S. 2018. Chitosan Applications in Food Industry. In: Biopolymers for Food Design. Academic Press. p. 469-491.

Marcotuli I, Colasuonno P, Hsieh YSY, Fincher GB, Gadaleta A. 2020. Non-Starch Polysaccharides in Durum Wheat: A Review. Int J Mol Sci. Apr 22;21:2933. Epub 2020/04/26.

Mellor DB, Stringer PA, Mountney GJ. 1958. The Influence of Glycogen on the Tenderness of Broiler Meat. Poultry Science. 1958/09/01/;37:1028-1034.

Mirhosseini H, Amid BT. 2012. A review study on chemical composition and molecular structure of newly plant gum exudates and seed gums. Food Research International. 2012/04/01/;46:387-398.

Morales V, Sanz ML, Olano A, Corzo N. 2006. Rapid Separation on Activated Charcoal of High Oligosaccharides in Honey. Chromatographia. 2006/08/01;64:1-6.

Oliveira DL, Wilbey RA, Grandison AS, Roseiro LB. 2015. Milk oligosaccharides: A review. International Journal of Dairy Technology.68:305-321.

Osemwegie OO, Adetunji CO, Ayeni EA, Adejobi OI, Arise RO, Nwonuma CO, Oghenekaro AO. 2020. Exopolysaccharides from bacteria and fungi: current status and perspectives in Africa. Heliyon. Jun;6:e04205. Epub 2020/06/25.

Padayachee A, Day L, Howell K, Gidley MJ. 2017. Complexity and health functionality of plant cell wall fibers from fruits and vegetables. Crit Rev Food Sci Nutr. Jan 2;57:59-81. Epub 2016/10/18.

Pandya U, Dhuldhaj U, Sahay NS. 2019. Bioactive mushroom polysaccharides as antitumor: an overview. Nat Prod Res. Sep;33:2668-2680. Epub 2018/05/05.

Pangestuti R, Arifin Z. 2018. Medicinal and health benefit effects of functional sea cucumbers. J Tradit Complement Med. Jul;8:341-351. Epub 2018/07/12.

Praveen MA, Parvathy KRK, Balasubramanian P, Jayabalan R. 2019. An overview of extraction and purification techniques of seaweed dietary fibers for immunomodulation on gut microbiota. Trends in Food Science & Technology. 2019/10/01/;92:46-64.

Saffer AM. 2018. Expanding roles for pectins in plant development. J Integr Plant Biol. Oct;60:910-923. Epub 2018/05/05.

Saha D, Bhattacharya S. 2010. Hydrocolloids as thickening and gelling agents in food: a critical review. J Food Sci Technol. Dec;47:587-597. Epub 2010/12/01.

Sato K, Nagai N, Yamamoto T, Mitamura K, Taga A. 2019. Identification of a Novel Oligosaccharide in Maple Syrup as a Potential Alternative Saccharide for Diabetes Mellitus Patients. Int J Mol Sci. Oct 11;20. Epub 2019/10/17.

Schmid J, Sieber V, Rehm B. 2015. Bacterial exopolysaccharides: biosynthesis pathways and engineering strategies. Front Microbiol. 2015-May-26;6:496. Epub 2015/06/16.

Sivam AS, Sun-Waterhouse D, Quek S, Perera CO. 2010. Properties of bread dough with added fiber polysaccharides and phenolic antioxidants: a review. J Food Sci. Oct;75:R163-174. Epub 2011/05/04.

Suryawanshi N, Jujjavarapu SE, Ayothiraman S. 2019. Marine shell industrial wastes–an abundant source of chitin and its derivatives: constituents, pretreatment, fermentation, and pleiotropic applications-a revisit. International Journal of Environmental Science and Technology. 2019/07/01;16:3877-3898.

Tanna B, Mishra A. 2019. Nutraceutical Potential of Seaweed Polysaccharides: Structure, Bioactivity, Safety, and Toxicity. Comprehensive Reviews in Food Science and Food Safety. 2019/05/01;18:817-831.

Thinh PD, Ly BM, Usoltseva RV, Shevchenko NM, Rasin AB, Anastyuk SD, Malyarenko OS, Zvyagintseva TN, San PT, Ermakova SP. 2018. A novel sulfated fucan from Vietnamese sea cucumber *Stichopus variegatus*: Isolation, structure and anticancer activity in vitro. Int J Biol Macromol. Oct 1;117:1101-1109. Epub 2018/06/10.

Usov AI. 2011. Chapter 4 - Polysaccharides of the red algae. In: Advances in Carbohydrate Chemistry and Biochemistry. Academic Press. p. 115-217.

Ustyuzhanina NE, Bilan MI, Dmitrenok AS, Nifantiev NE, Usov AI. 2018. Fucosylated chondroitin sulfates from the sea cucumbers *Holothuria tubulosa* and *Holothuria stellati*. Carbohydr Polym. Nov 15;200:1-5. Epub 2018/09/05.

Villarruel-López A, Ascencio F, Nuño K. 2017. Microalgae, a Potential Natural Functional Food Source – a Review. Pol J Food Nutr Sci. 2017;67:251-263.

Vinogradov E, Petersen BO, Duus J, Wasser S. 2004. The structure of the glucuronoxylomannan produced by culinary-medicinal yellow brain mushroom (Tremella mesenterica Ritz.:Fr., Heterobasidiomycetes) grown as one cell biomass in submerged culture. Carbohydr Res. Jun 1;339:1483-1489. Epub 2004/06/05.

Wu R-Q, Li Z-X, Yang J-P, Xing X-H, Shao D-Y, Xing K-L. 2009. Mutagenesis induced by high hydrostatic pressure treatment: a useful method to improve the bacterial cellulose yield of a Gluconoacetobacter xylinus strain. Cellulose. 2010/04/01;17:399-405.

Wu YJ, Wei ZX, Zhang FM, Linhardt RJ, Sun PL, Zhang AQ. 2019. Structure, bioactivities and applications of the polysaccharides from *Tremella fuciformis* mushroom: A review. Int J Biol Macromol. Jan;121:1005-1010. Epub 2018/10/21.

Xiong Q, Song Z, Hu W, Liang J, Jing Y, He L, Huang S, Wang X, Hou S, Xu T, et al. 2020. Methods of extraction, separation, purification, structural characterization for polysaccharides from aquatic animals and their major pharmacological activities. Crit Rev Food Sci Nutr. 2020/01/02;60:48-63. Epub 2018/10/05.
